# Denitrification in human dental plaque

**DOI:** 10.1186/1741-7007-8-24

**Published:** 2010-03-22

**Authors:** Frank Schreiber, Peter Stief, Armin Gieseke, Ines M Heisterkamp, Willy Verstraete, Dirk de Beer, Paul Stoodley

**Affiliations:** 1 Microsensor Research Group, Max-Planck-Institute for Marine Microbiology, Bremen, Germany; 2 Laboratory of Microbial Ecology and Technology (LabMET), Ghent University, Ghent, Belgium; 3 Center for Genomic Sciences, Allegheny General Hospital/Allegheny-Singer Research Institute, Pittsburgh, PA, USA; 4 National Centre for Advanced Tribology at Southampton (nCATS), School of Engineering Sciences, University of Southampton, Southampton, UK

## Abstract

**Background:**

Microbial denitrification is not considered important in human-associated microbial communities. Accordingly, metabolic investigations of the microbial biofilm communities of human dental plaque have focused on aerobic respiration and acid fermentation of carbohydrates, even though it is known that the oral habitat is constantly exposed to nitrate (NO_3_^-^) concentrations in the millimolar range and that dental plaque houses bacteria that can reduce this NO_3_^- ^to nitrite (NO_2_^-^).

**Results:**

We show that dental plaque mediates denitrification of NO_3_^- ^to nitric oxide (NO), nitrous oxide (N_2_O), and dinitrogen (N_2_) using microsensor measurements, ^15^N isotopic labelling and molecular detection of denitrification genes. *In vivo *N_2_O accumulation rates in the mouth depended on the presence of dental plaque and on salivary NO_3_^- ^concentrations. NO and N_2_O production by denitrification occurred under aerobic conditions and was regulated by plaque pH.

**Conclusions:**

Increases of NO concentrations were in the range of effective concentrations for NO signalling to human host cells and, thus, may locally affect blood flow, signalling between nerves and inflammatory processes in the gum. This is specifically significant for the understanding of periodontal diseases, where NO has been shown to play a key role, but where gingival cells are believed to be the only source of NO. More generally, this study establishes denitrification by human-associated microbial communities as a significant metabolic pathway which, due to concurrent NO formation, provides a basis for symbiotic interactions.

## Background

The human body is naturally colonised by a diverse array of micro-organisms whose metabolic activity is important for human physiology and health [1]. Most studies that assess the functional potentials and controls of these complex communities rely on: (i) the characterisation of individual isolates or enrichments, (ii) quantification of micro-organisms that are thought to mediate a certain process, or (iii) metagenomic analysis of a certain body region. Established methods of microbial ecology that allow the direct measurement of metabolic conversions in natural microbial samples from humans under different experimental conditions, such as incubation with isotopically-labelled substrates, dye probes for specific compounds combined with microscopy or electrochemical microsensors, are rarely reported. However, different microbial pathways, including fermentation, sulfate reduction, methanogenesis and acetogenesis [2-5], have been proposed to occur in humans. Surprisingly, denitrification (the respiratory reduction of nitrate (NO_3_^-^) or nitrite (NO_2_^-^) via nitric oxide (NO) to nitrous oxide (N_2_O) or dinitrogen (N_2_) [6]) is believed to be insignificant in human-associated microbial communities [7], even though NO_3_^- ^and NO_2_^- ^co-occur in significant concentrations with micro-organisms in various body regions, such as the human oral cavity [8].

Denitrification is performed by facultative anaerobic micro-organisms and is coupled to the oxidation of reduced organic carbon or reduced inorganic compounds, such as ferrous iron, hydrogen sulfide or hydrogen [6,9]. The reductive sequence (NO_3_^- ^> NO_2_^- ^> NO > N_2_O > N_2_) of denitrification is mediated by periplasmic and membrane-bound enzymes specific for each step. The most important genes for the detection of denitrification in complex microbial samples are *narG *for NO_3_^- ^reductase, *nirS *and *nirK *for NO_2_^- ^reductases, *qnorB *or *cnorB *for NO reductases, and *nosZ *for N_2_O reductase. Denitrifying bacteria release NO or N_2_O as intermediates during metabolic activity in pure culture [10,11] and in complex microbial communities, such as soils [12], nitrogen cycling biofilms [13] and ingested bacteria within different invertebrates guts [14,15].

Notably, human saliva contains NO_3_^- ^concentrations in the millimolar range, because dietary NO_3_^- ^is concentrated in salivary glands after it is absorbed from the intestine into the blood [8]. Thus, the human-associated microbial biofilm community of dental plaque and bacteria that cover other oral surfaces are exposed to NO_3_^-^. However, investigations of plaque metabolism have focused on aerobic respiration and acid fermentation of carbohydrates [16]. Experiments with rat tongues as well as tooth and other surfaces in the human mouth have shown that salivary NO_3_^- ^can be converted by oral micro-organisms to NO_2_^-^, explaining the presence of NO_2_^- ^in addition to NO_3_^- ^in saliva [17,18]. Detection of NO in air incubated in the human mouth has led to the hypothesis that bacterially-derived salivary NO_2_^- ^is chemically reduced to NO in acidic microenvironments in the oral cavity [18,19]. The underlying processes have never been directly demonstrated because NO could not be measured in dental biofilms over relevant spatial scales. Therefore, other investigators considered NO_2_^- ^in human saliva a stable oxidation product of NO synthase-derived NO that is produced by gingival cells to regulate the gum immune and vascular systems [20,21].

Due to the possible formation of NO, plaque nitrogen metabolism might be important to dental health. Dental plaque causes periodontal diseases and dental caries, affecting almost every human being [22,23]. As an inflammatory disorder of gum tissue surrounding the teeth, periodontal diseases might be especially affected by nitrogen metabolism of dental plaque, if NO is generated as a side product at the gum-plaque interface. NO plays a complex, but not well understood role in periodontal diseases [24,25]. NO, at low concentrations, is an important signalling molecule that coordinates functions of immune system cells that are involved in inflammatory processes [26]. Bacterial lipopolysaccharides stimulate production of proinflammatory cytokines, which induce production of high, cytotoxic NO concentrations by certain immune system cells. Furthermore, high NO levels during inflammation induce expression of matrix metalloproteinases in neutrophiles, which mediate soft tissue degradation [27].

Besides its potential importance to dental health, oral nitrogen metabolism is important for human physiology [8]. The formation of NO_2_^- ^as a denitrification intermediate by oral micro-organisms leads to chemical conversion of NO_2_^- ^to NO in the acidic stomach, acting as an antimicrobial agent against pathogenic bacteria and stimulating gastric blood flow. Moreover, NO_2_^- ^is absorbed into plasma, where it serves as a NO source for the regulation of vasodilatation under hypoxic conditions [8]. It is still unclear whether microbial nitrogen metabolism in human dental plaque is significant in comparison to other oral surfaces.

In the present study, we hypothesise that dental plaque represents a habitat for microbial denitrification in humans, driving the biological conversion of salivary NO_3_^- ^to the denitrification intermediates NO and N_2_O, and to the final product N_2_. We use direct microbial ecology methods, including a recently developed NO microsensor [28], to demonstrate *in situ *NO formation during denitrification in dental plaque and to show that NO is formed at concentrations that are significant for signalling to host tissue. In addition, we aim to show the *in vivo *significance of plaque denitrification for the formation of denitrification intermediates by correlating the oral accumulation of N_2_O in humans to salivary NO_3_^-^/NO_2_^- ^concentrations and to the presence of plaque.

## Results

### Dental plaque mediates aerobic denitrification

**Figure 1:**
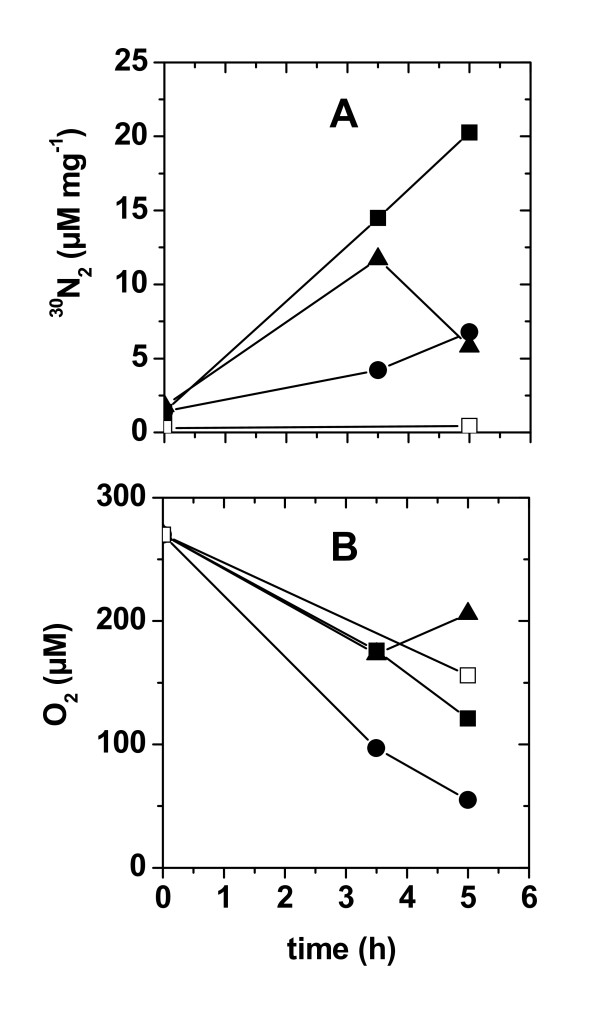
**Denitrification in dental plaque**. Dental plaque of three individuals was suspended in aerobic mineral medium buffered at pH 7.2 containing 2% sucrose and 50 μM Na^15^NO_3_. Formation of **(a) **^30^N_2 _(in μM/mg protein) and **(b) **apparent O_2 _concentrations (in μM) were measured in a time series experiment. Each symbol type represents ^30^N_2 _and O_2 _measurements of dental plaque incubations from one individual. Control measurements were done in the absence of Na^15^NO_3 _(open symbols).

Dental plaque converted NO_3_^- ^to N_2 _by denitrification. This was shown by ^30^N_2 _formation from ^15^NO_3_^- ^during incubation of dispersed dental plaque (Figure 1a). The occurrence of complete denitrification in dental biofilms was corroborated by polymerase chain reaction (PCR) detection of all genes (NO_3_^- ^reductase, NO_2_^- ^reductase, NO reductase, N_2_O reductase) that are necessary for the respiratory reduction of NO_3_^- ^to N_2 _(Table 1). Genes for respiratory NO reductases were restricted to the presence of the quinol-dependent type (*qnorB*), but not of the cytochrome *c*-dependent type (*cnorB*).

**Table 1:** Denitrification genes in dental biofilms of five volunteers

Volunteer	NO_3_^- ^reductase	NO_2_^- ^reductase		NO reductase		N_2_O reductase
	
	*narG*	*nirS*	*nirK*	*cnorB*	*qnorB*	*nosZ*
A	+	+	+	-	+	+

B	+	+	+	-	+	+

C	+	+	+	-	+	+

D	+	-	+	-	+	+

E	+	NA	NA	-	+	NA

Results are based on detection of a PCR product with the expected size or on the additional analysis of the sequence of the PCR product.

NA = not analysed.

**Figure 2:**
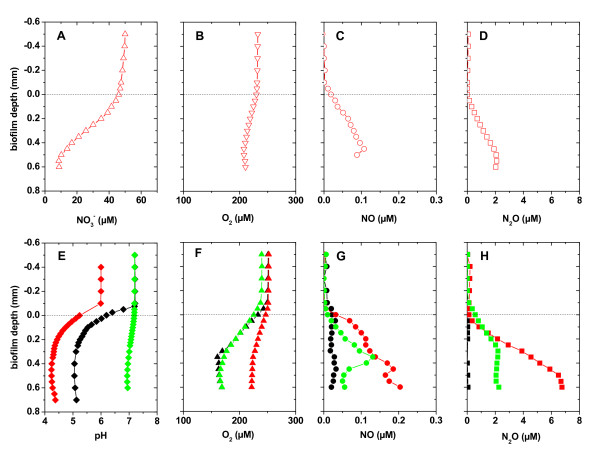
***In situ *detection of metabolic activity and microenvironmental conditions in a dental biofilm outside the mouth**. Microsensors were used to measure concentration profiles of NO_3_^-^, O_2_, NO, N_2_O and pH in dental plaque. The medium contained a non-buffered mineral mix and 2% sucrose. The upper panels **(a-d) **show measurements with 50 μM NaNO_3 _(open red symbols) in the overlying medium. The lower panels **(e-h) **show measurements in the absence of NaNO_3 _(black symbols) and in the presence of 760 μM NaNO_3 _(red and green symbols). Measurements depicted by the green symbols were performed in the presence of phosphate-buffered saline (pH 7.2) and 760 μM NaNO_3_. The horizontal line represents the biofilm surface. Measurements were done in the same sample spot and thus are directly comparable.

Two lines of evidence suggested that denitrification in dental biofilms occurred under aerobic conditions. First, we observed ^30^N_2 _production from plaque that was suspended in air-saturated medium amended with 50 μM ^15^NO_3_^- ^(Figure 1a). O_2 _measurements in this medium showed that aerobic heterotrophic respiration did not lead to anoxic conditions during the incubation period (Figure 1b). Second, microsensor measurements showed that NO_3_^- ^was consumed in the presence of O_2 _and that also the denitrification intermediates NO and N_2_O were formed in the presence of O_2 _(Figure 2a-d). At this low NO_3_^- ^concentration it is conceivable that all NO_3_^- ^was used for assimilation into biomass, and was thus not available for respiratory denitrification. However, in this plaque sample NO_3_^- ^was not completely consumed (that is, NO_3_^- ^was not limiting) when present at a concentration of 50 μM NO_3_^- ^(Figure 2a). Thus, NO_3_^- ^assimilation and denitrification must have been already present at their maximum capacity at 50 μM NO_3_^-^. Further increases of the NO_3_^- ^concentration to 760 μM will most likely not change the contribution of both pathways to the total NO_3_^- ^uptake. In turn, the biofilm remained oxic when subjected to 760 μM NO_3_^- ^and produced the denitrification intermediates NO and N_2_O (Figure 2f-h) indicating that aerobic denitrification was also active at high NO_3_^- ^concentrations.

### Chemical and biological NO and N_2_O formation during plaque denitrification is pH dependent

**Figure 3:**
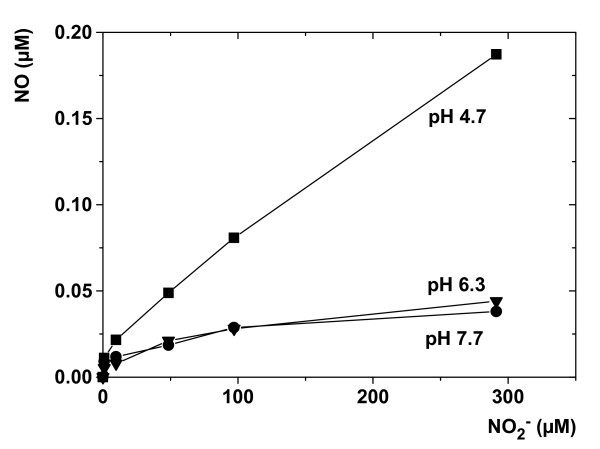
**Chemical formation of NO by acidic decomposition of NO_2_^-^**. NO formation was measured with a NO microsensor during titration of increasing NO_2_^- ^concentrations in different solutions of phosphate-buffered saline at varying pH.

NO_3_^- ^was the source for NO and N_2_O in dental biofilms. This was shown by NO and N_2_O formation being restricted to the presence of NO_3_^- ^(Figure 2g, h). NO formation in dental biofilms was mediated by both biological NO_2_^- ^reduction and presumably acidic decomposition of NO_2_^-^. Biological NO_2_^- ^reduction was the sole process that produced NO when the medium was buffered at approximately pH 7. In non-buffered medium, bacterial activity decreased biofilm pH < 5 (Figure 2e) and depth-averaged NO concentrations increased from 0.08 to 0.15 μM (Figure 2g). Titration of 50 μM NO_2_^- ^to a buffer at pH 4.7 showed that acidic decomposition of NO_2_^- ^caused chemical formation of approximately 0.05 μM NO (Figure 3), which is in the same range than the observed increase in the biofilm at pH < 5. It is already known that plaque can form NO_2_^- ^by NO_3_^- ^reduction [17]. NO_2_^- ^can also naturally accumulate in saliva to concentrations of 50 μM and higher (data not shown, and [18]). Taken together, this suggests that acidic decomposition of NO_2_^- ^contributes to NO formation at low plaque pH levels, while biological NO formation may still occur in parallel.

The absolute increase of NO due to acidic conditions was small from the perspective of the metabolic homeostasis of denitrification. This was evident because depth-averaged increases of N_2_O, the product of NO reduction, were approximately two orders of magnitude higher than those of NO concentrations under acidic conditions. This suggests that biofilm bacteria efficiently convert most NO to N_2_O and thereby keep the steady-state concentration of cytotoxic NO low, as has been also observed in environmental biofilms [13].

### NO formation decreases O_2 _uptake of dental plaque

Oxygen uptake in the presence of NO_3_^- ^was higher at neutral pH than under acidic conditions (Figure 2e, f). The O_2 _profiles showed that the flux of O_2 _decreased by 50%, namely from -105 nmol/cm^2^/h under buffered conditions to -43 nmol/cm^2^/h under non-buffered conditions. Acidic pH alone did not lead to reduced O_2 _uptake when NO_3_^- ^was absent, as the O_2 _flux was -143 nmol/cm^2^/h. Decreased bacterial O_2 _consumption might result from direct toxic effects of the highest NO concentration (0.15 to 0.2 μM), such as binding of NO to terminal, respiratory O_2 _reductases [29]. However, the absolute increase from 0.08 to 0.2 μM may not affect respiration as concentrations above 0.8 μM were previously shown to be necessary to inhibit O_2 _reduction in *Escherichia coli *[30]. In addition, instead of facilitating O_2 _reduction, a small fraction of electrons might be used preferentially for detoxification of NO by reduction to N_2_O, contributing to increased N_2_O concentrations and inhibited O_2 _uptake (Figure 2f, h).

### N_2_O production in the human mouth is dependent on salivary NO_3_^- ^and on the presence of dental plaque

**Figure 4:**
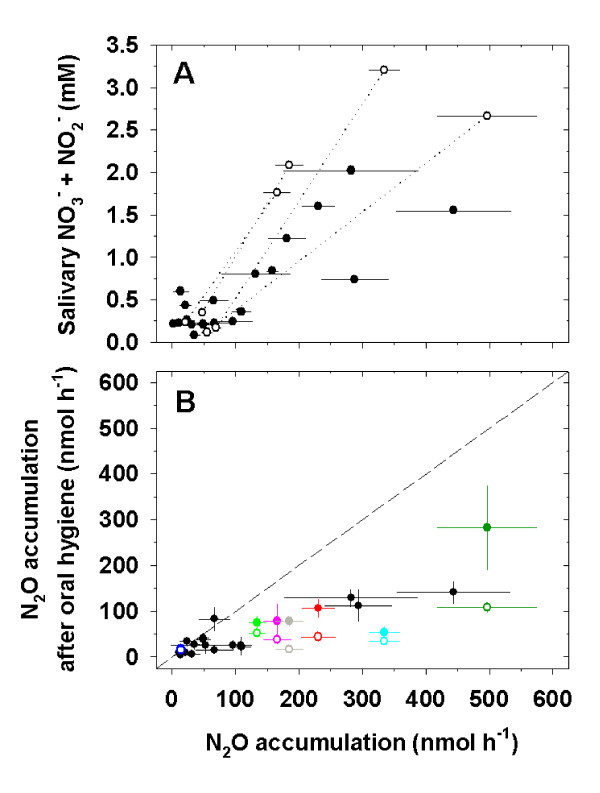
**N_2_O formation in the human mouth is dependent on salivary NO_2_^-^/NO_3_^- ^concentrations and the presence of dental plaque**. **(a)** Correlation of oral N_2_O production and salivary NO_2_^-^/NO_3_^- ^concentration in 15 volunteers with unbrushed teeth. Each data point represents the rate of oral N_2_O accumulation of one individual on a certain day (black circles). Some volunteers were sampled on more than 1 day resulting in 19 data points in total. Four volunteers were additionally sampled before and after drinking NO_3_^-^-rich beetroot juice to increase salivary NO_2_^-^/NO_3_^- ^concentration and oral N_2_O accumulation (white circles connected by dotted line). **(b) **Effect of oral hygiene on N_2_O accumulation rate in the mouth. Oral N_2_O accumulation rate of individuals before tooth brushing plotted against the N_2_O accumulation rate after tooth brushing (closed circles). In six individuals an antiseptic mouth rinse that affects bacteria in the entire oral cavity was applied after tooth brushing (open circles, each of the six individuals is represented by a unique colour). For example, an individual (dark green) with an oral N_2_O accumulation rate of 500 nmol/h reduced the rate to 290 nmol/h by tooth brushing. Subsequent application of a mouth rinse resulted in a rate of 110 nmol/h. The dashed line corresponds to the absence of an effect of oral hygiene on the oral N_2_O accumulation. The error bars indicate the standard error of five replicate measurements of the oral N_2_O accumulation rate.

We incubated air in the human mouth ('mouth air') and measured the rate of N_2_O accumulation to quantify the *in vivo *significance of denitrification in the oral habitat. We related N_2_O accumulation in mouth air to the presence of dental biofilms and salivary NO_3_^-^/NO_2_^- ^concentrations (Figure 4). N_2_O accumulation in the presence of dental plaque varied strongly between the subjects and ranged from 11 to 443 nmol/h. N_2_O accumulation between subjects increased with increasing salivary NO_3_^-^/NO_2_^- ^concentrations (Figure 4a). Drinking 200 ml beetroot juice that contained 12 mmol/l NO_3_^- ^increased the salivary NO_3_^-^/NO_2_^- ^concentrations, which led to an increase of between 3.8 and 9.1 fold in the rate of oral N_2_O accumulation.

Dental biofilms were the main sites of N_2_O production in the human mouth. This was evident because the combined application of ordinary tooth brushing with an antiseptic mouthwash decreased oral N_2_O accumulation rate by 82%, while tooth brushing alone decreased the rate of oral N_2_O accumulation by 62% (Figure 4b).

## Discussion

**Figure 5:**
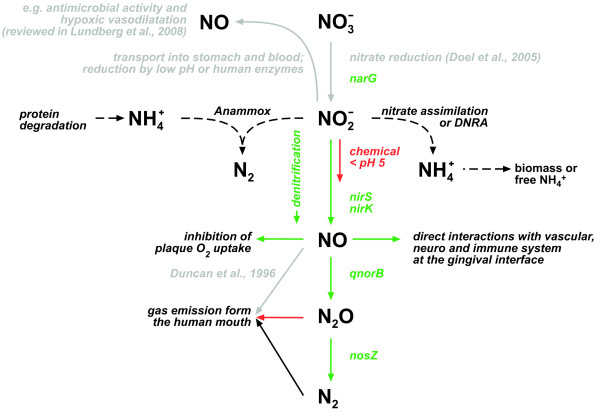
**Microbial conversions of salivary NO_3_^- ^in human dental plaque and its possible consequences**. Grey lines show pathways that have been reported previously. Dotted lines show potential pathways that have not been reported to occur in dental plaque in this or other studies. Coloured lines show pathways that are suggested to occur by this study. Green lines show biologically-mediated pathways and red lines show chemically-mediated or physically-mediated pathways. Genes encoding for enzymes that mediate individual steps of denitrification are depicted if detected in dental biofilms via polymerase chain reaction (PCR). Genes of the cytochrome *c*-dependent NO reductase (cNorB) were not detected. Anammox = anaerobic oxidation of ammonium; DNRA = dissimilatory nitrate reduction to ammonium.

Our data show unambiguously that denitrification is a relevant process in a human associated microbial community. Until now, it was assumed that complete NO_3_^- ^reduction in humans is restricted to the dissimilatory nitrate reduction to ammonium (DNRA), because most bacterial isolates from humans are able to perform this reaction [7,31]. DNRA is known to be a strictly anaerobic process that is favoured over denitrification in anaerobic, reduced environments [32]. Accordingly, DNRA might prevail in the reduced, anaerobic environment of the human gut, while denitrification is present in the more oxidised dental plaque. In turn, it is conceivable that DNRA is present in plaque that is recalcitrant to removal and thus, constantly anoxic and more reduced. Theoretically, in such biofilms NO_2_^- ^reduction might be coupled to the anaerobic oxidation of ammonium (anammox) (Figure 5, black dotted lines), especially if protein degradation of host tissue or DNRA could provide a source for ammonium, as has been reported in marine open water habitats [33]. Denitrification, however, might be present in other oxidised environments in humans where bacteria and NO_3_^- ^co-occur. For example, NO_3_^- ^is also present in other body fluids than saliva that may provide a rather oxidised environment (for example, blood 20 to 40 μM and urine approximately 500 μM) [8]. Thus, denitrification might be relevant in microbial biofilms that are associated to other diseased or healthy sites, such as cystic fibrosis lungs, otitis media ears, implants, catheters and vaginal mucosa [34].

Denitrification and DNRA are fundamentally different with respect to their final products, metabolic controls and released intermediates resulting in different effects on host physiology. Ammonium, the final product of DNRA, is available to host cells and associated microbes as a form of fixed nitrogen. Moreover, DNRA in humans may accumulate ammonium to detrimental concentrations [35]. In contrast, N_2_, the final product of denitrification, represents a loss of fixed nitrogen from the host and does not affect human cells. Furthermore, production of the signalling molecule NO by microbial denitrification might shape the interactions between the host cells and their associated microbial community.

Interestingly, denitrification in dental biofilms occurred under aerobic conditions. This shows that dental plaque does not necessarily have to display anoxic microsites for denitrification to occur. The ability to denitrify in the presence of O_2 _has been observed for isolated bacterial strains and occasionally for microbial communities [36,37]. Aerobic denitrification guarantees a stable electron accepting process in a NO_3_^-^-rich habitat exposed to frequent fluctuations in O_2 _concentration without energy-demanding expression of new enzyme systems [38]. This may perfectly apply to the oral habitat that is characterised by high salivary NO_3_^- ^and potentially fluctuating O_2 _concentrations in the mouth.

Despite the microbial diversity of dental biofilms [16], we could only detect genes for respiratory NO reductases of the quinol-dependent type (*qnorB*), but not of the cytochrome *c*-dependent type (*cnorB*). Interestingly, genes that code for qNorB are also found in non-denitrifying, pathogenic bacteria, where it contributes to NO detoxification, instead of respiratory electron transport [39]. Apparently, respiratory NO reduction is exclusively mediated by qNorB in the investigated dental biofilms. Additionally, qNorB might be used as a protective enzyme against toxic NO derived from host cells, acidic decomposition of NO_2_^- ^and other biofilm bacteria.

Our results allowed us to formulate a mechanistic model for nitrogen conversions in dental plaque (Figure 5). First, reduction of salivary NO_3_^- ^leads to the formation of NO_2_^- ^[17], which is further denitrified to form the intermediates NO and N_2_O and finally N_2 _(Figure 5, green lines). The mechanistic model involves a pH-controlled chemical conversion step from NO_2_^- ^to NO in addition to the biological conversion step (Figure 5, red line). The chemical conversion of NO_2_^- ^to NO occurs if acid fermentation decreases plaque pH < 5. High turnover of NO under acidic conditions leads to decreased O_2 _uptake in dental biofilms. This argues toward an NO-mediated metabolic coupling of different microbial activities in dental plaque. Moreover, bacteria respond to NO as a signalling molecule. Specifically, NO is involved in the dispersal of bacteria from biofilms [40]. Together this makes NO a possible bacterially-derived factor that balances the development of a natural dental plaque community. Thus, NO might be an important factor from the perspective of the 'ecological plaque hypothesis', which states that environmental factors (for example, pH and salivary NO_3_^-^) in the mouth determine if the dental plaque microbial community is dominated by either health-sustaining or disease-causing micro-organisms [41].

Human cells can produce NO from arginine by NO synthase activity and respond to NO as a signal molecule produced by other cells [26]. In gingival tissue, NO is known to be involved in blood pressure regulation and in inflammatory processes, such as those in periodontal diseases [24,27]. Under acidic conditions, the depth-averaged NO concentration in dental plaque increased from 0.08 to 0.15 μM (Figure 2g), which is in a physiological effective range [42,43] for local blood pressure regulation, neurosignalling events and immune system modulation in tissues close to plaque. Hence, we hypothesise that pH fluctuations and plaque denitrification may locally affect blood flow, signalling between nerves, and inflammatory processes in the gum by modulating the concentration of NO (Figure 5, green line). NO-mediated interactions will be different in cariogenic as compared to periodontal plaque, because both are characterised by distinct pH regimes [41]. While low pH levels in cariogenic plaque may induce chemical NO formation leading to high NO concentrations, NO formation in periodontal plaque will be restricted to microbial processes, because it is characterised by pH levels > 7. As discussed above, microbial denitrification might be relevant in other healthy and diseased sites of the body. Thus more generally, microbial denitrification might be considered an alternative route for NO formation in humans and provides a basis for symbiotic interactions between human-associated microbes and adjacent host cells.

The presence of dental plaque caused accumulation of N_2_O, as an intermediate of denitrification, in mouth air depending on salivary NO_3_^- ^concentrations. This demonstrates that denitrification occurs *in vivo *and that dietary NO_3_^-^-uptake influences plaque denitrification. The average rate of oral N_2_O emission from 15 volunteers with unbrushed teeth and non-manipulated salivary NO_3_^-^/NO_2_^- ^concentrations was 80 nmol/h. While earlier investigations of the breath air of human beings revealed N_2_O concentrations above the ambient atmospheric level [44-46], our study presents the first oral-associated emission rates of this greenhouse gas [47] by humans. Extrapolating our data to the world population of currently 6.7 billion people, oral-associated N_2_O emission by humans is 0.00013 Tg N/a, representing an insignificant amount of 0.0008% of the total, annual N_2_O emission of 16.4 Tg N/a to the atmosphere [48].

Dental biofilms were the main sites of N_2_O production in the human mouth. This result and the microsensor data strongly suggest that dental plaque is also the main site for oral formation of the other denitrification intermediates NO_2_^- ^and NO. Thus, NO_2_^- ^measurements in saliva [20,21] are not an adequate proxy for NO formation by human host cells in the mouth (Figure 5). Moreover, plaque denitrification needs to be considered when using NO and NO_2_^- ^measurements in breath and saliva to diagnose systemic diseases, such as renal failure [49,50] and cystic fibrosis [51]. More generally, the importance of dental plaque for the formation of denitrification intermediates as compared to other oral surfaces indicates that plaque bacteria are as important for oral nitrogen conversions than bacteria located on other oral surfaces [17]. Consequently, dental biofilms and salivary NO_3_^- ^concentrations play a crucial role in the regulation of body NO_2_^- ^concentration and affect NO_2_^-^-related physiological functions in the human body, such as hypoxic vasodilatation and antimicrobial activity in the acidic stomach (Figure 5, grey lines) [8].

Numerous anatomical sites, including the skin, mouth, stomach, colon, and vagina, are inhabited by distinct microbial communities, which are characterised by a large diversity. This suggests a versatile potential of different metabolic pathways mediated by micro-organisms that affect human physiology. However, activities or the functional potentials of complex human-associated microbial communities have rarely been investigated [1]. Thus, we anticipate that investigations of human-associated microbial communities with techniques that measure their *in situ *activities will lead to the discovery of unexpected functions and interactions between microbes and humans if expanded to elemental cycles of carbon, sulfur, iron and others. The microbial ecology toolbox available for these experiments comprises techniques, such as microsensors, and isotopic or radioactive labelling with detection in bulk medium and on a single cell level, all of which can be combined with phylogenetic identification [52-55]. This ecophysiological approach will allow the direct testing of hypotheses that emerge from metagenomic data that is generated in the context of the human microbiome project [56].

## Conclusions

Human dental plaque mediates denitrification based on salivary NO_3_^-^. Plaque denitrification is performed under aerobic conditions and leads to biological and chemical NO and N_2_O formation in a pH-dependent manner. Plaque O_2 _uptake is inhibited by NO. Thus, formation of NO mediates metabolic interactions between plaque bacteria. Moreover, NO is produced at concentration levels that allow signalling to human host cells, pointing toward a significant role in the regulation of inflammatory disorders of gum tissue (that is, periodontal diseases). Microbial denitrification is, besides NO synthase activity and acidic decomposition of NO_2_^-^, an alternative pathway of NO formation in humans. Denitrification in dental plaque is a relevant site for production of intermediates of nitrogen cycling in the human mouth and is dependent on salivary NO_3_^-^. Thus, breath analysis for the detection of systemic diseases should consider plaque activity, and denitrification in dental plaque may affect the physiology of the whole human body.

## Methods

### Samples

Samples of natural, dental plaque were obtained with toothpicks or dental floss from male and female volunteers (25 to 52 years in age), who had not taken antibiotics, and not being diagnosed as having periodontitis and/or other severe diseases. Experiments involving human samples were approved by the Federal Dentists Chamber Bremen (Landeszahnärztekammer Bremen, Germany) and all volunteers gave their written consent.

### N2 production from isotopically-labelled NO_3_^-^

Dental biofilms were collected with a toothpick from dental surfaces and interproximal (IP) spaces of three volunteers and were washed twice in phosphate-buffered saline (PBS; pH 7.2). The protein content of the sample was determined after Lowry [57]. Biofilms were homogenised by vortexing, the material of each individual was equally distributed to three exetainers (3.8 ml) and filled with air saturated incubation medium (phosphate buffered saline and 2% sucrose). The incubation was immediately started by adding 50 μM Na^15^NO_3 _and was performed under continuous mixing at 37°C. Dissolved O_2 _concentration was measured with an O_2 _microsensor, directly before biological reactions were stopped by adding ZnCl_2 _to a final concentration of 0.5% at three time points (t_0 _= 0 h; t_1 _= 3.5 h; t_2 _= 5 h). A quadrupole mass spectrometer (GAM 200, IP Instruments, Bremen, Germany) was used to measure ^30^N_2 _after introducing a 2 ml helium headspace into the closed exetainer and equilibration between the liquid and gas phase.

### Microsensor measurements

Plaque from two volunteers was subjected to *in situ *measurements with NO, N_2_O, O_2_, pH and NO_3_^- ^microsensors outside the mouth. Biofilms were carefully recovered with toothpicks or dental floss from the IP spaces of the upper or lower molars by volunteers that did not brush their teeth for at least 24 h. Whole biofilm pieces were placed on solid agar (1.5%), fixed with a drop of molten agar (0.5%) and covered with non-buffered sucrose/salt medium (68 mM NaCl, 8 mM MgCl_2_, 3.6 mM CaCl_2_, 26.8 mM KCl, 2% sucrose; pH 6.6 to 7.2). Biofilms equilibrated for at least 20 min before the measurements, which were performed within 6 to 8 h after biofilm retrieval. Manufacturing of amperometric NO, N_2_O and O_2_, and ion-selective pH and NO_3_^- ^microsensors [28,58-60] and microsensor measurements [28] were conducted as previously described. Steady state microprofiles were measured before and after 760 μM NaNO_3 _was added, while an air jet directed on the medium surface created a constant flow regime above the biofilm. To investigate nitrogen cycling at pH 6 to 7 in the biofilm the medium was supplemented with phosphate buffer (10 mM Na_2_HPO_4_, 1.8 mM KH_2_PO_4_, pH 7.2; resembling the concentration in 1 × PBS), thereby excluding chemical NO_2_^- ^reduction. To increase sensor performance, NO_3_^- ^microprofiles were measured in medium with lower salt content, and in the presence and absence of 50 μM NaNO_3_, instead of 760 μM. All presented measurements were performed in the same biofilm spot. Thus, the measurements are suitable to draw mechanistic conclusions. However, the data do not account for biofilm heterogeneity and are not suitable for calculation of average fluxes over a given biofilm surface. We repeated the same experiment with a biofilm from a second individual, which essentially showed the same treatment effects (see Additional file 1, Figure S1 and supplementary discussion).

### Molecular analysis of denitrification genes from dental biofilms

Dental plaque was collected from dental surfaces and IP spaces with sterile toothpicks by five volunteers that had not brushed their teeth or eaten for 12 h. DNA was extracted according to a protocol optimised for dental plaque [61]. PCR amplification of partial sequences of the denitrification genes *narG*, *nirS*, *nirK*, *cnorB*, *qnorB*, and *nosZ *was performed in a total volume of 20 μl containing 2 μl of 10 × PCR buffer, 250 μM of each deoxyribonucleoside triphosphate, 1 U of *Taq *polymerase (5 Prime GmbH, Hamburg, Germany), 0.3 mg/ml bovine serum albumin (BSA), 0.5 μM of each primer and 10 to 100 ng DNA. Published primers that target a wide spectrum of denitrification genes from different organisms were used and PCR experiments were performed as described in the corresponding protocols with some modifications (Additional file 1, Table S1). Amplicons were analysed by electrophoresis on 1% agarose gels and subsequent ethidium bromide staining. For 11 out of 22 amplicons with the expected size clone libraries were constructed and sequenced to confirm that PCR products corresponded to the targeted genes. Amplicons were purified with the QIAQuick PCR purification kit (Qiagen, Hilden, Germany) and cloned using the TOPO TA cloning system (Invitrogen, Carlsbad, CA, USA) following the manufacturer's instructions. The obtained sequences were analysed with BLAST http://www.ncbi.nlm.nih.gov. Only 1 of the 11 clone libraries did not contain the targeted gene (nirS, subject D).

### N_2_O accumulation in mouth air

A total of 15 volunteers (25 to 52 years in age) were asked not to brush their teeth the night and morning before the measurement. They were allowed to eat and drink, but not during the last hour before the measurements. To exclusively measure N_2_O that is produced in the mouth, but not in the lung or the stomach, we injected ambient air (30 ml) into the empty mouth. Subsequently, volunteers were asked to breathe through the nose with the mouth closed off from the nasopharynx and keep the injected air in their mouth. We defined this air as mouth air in which orally-produced N_2_O accumulated. Two gas samples (1 ml) were withdrawn through the blunt canula of a syringe after 30 and 90 s and filled into gas-tight exetainers (3 ml). This sampling scheme was repeated five times with teeth unbrushed and five times with teeth brushed by the volunteers. The N_2_O accumulation rate of seven volunteers was additionally measured after both teeth brushing and a 1-min antiseptic mouthwash that contains chlorhexidine, following the package insert (Chlorhexamed fluid, 0.1%, GlaxoSmithKline, Bühl, Germany). Before brushing the teeth, the volunteers collected 1 ml of saliva that was immediately frozen for later analysis of the NO_3_^-^/NO_2_^- ^concentration. Subsamples of mouth air were analysed for N_2_O concentration using a gas chromatograph with a ^63^Ni electron capture detector (Agilent GC7890, Agilent Technologies, Waldbronn, Germany). From the concentration difference between 30 and 90 s and the incubated volume of air, the rate of N_2_O accumulation was calculated in nmol/individual/h. The increase of N_2_O concentration in mouth air was shown to be linear for at least 240 s in additional test runs.

In a separate experiment, the N_2_O accumulation rate of four volunteers with teeth unbrushed was determined before and 2 h after drinking 200 ml of beetroot juice that contained 12 mM NO_3_^-^. The volunteers collected 0.5 ml of saliva before and then hourly after drinking the beetroot juice for later analysis of the NO_3_^-^/NO_2_^- ^concentration. Maximum salivary NO_3_^- ^and NO_2_^- ^concentrations were generally measured 2 h after drinking beetroot juice. Saliva samples were cleared by centrifugation and then analysed for NO_3_^-^/NO_2_^- ^with the VCl_3 _reduction method [62] followed by NO measurement on a chemiluminescence detector (CLD 86, EcoPhysics, Duernten, Switzerland).

### Data deposition

Sequences for the denitrification genes obtained in this study have been submitted to the EMBL Nucleotide Sequence Database under the accession numbers FN401446 to FN401486

## Authors' contributions

PSto, AG and DdB initiated the study, performed initial microsensor measurements and formulated, together with WV, a conceptual framework for nitrate conversions in dental plaque. Microsensor measurements were designed by PSto and FS, and performed and analysed by FS. Isotopic labelling was designed, performed and analysed by PSti and FS. Experiments for oral N_2_O emission were designed by PSti and FS, and performed and analysed by PSti. Molecular analysis of dental plaque was performed by AG and IH, and analysed by IH and PSti. PSti and FS wrote the manuscript with input from PSto and DdB.

## Supplementary Material

Additional file 1 **Supplementary information**. Figure S1, discussion of Figure S1, and Table S1. Click here for file
