# Supplementary material for: Denitrification in human dental plaque
Source: BMC Biol. 2010 Mar 22;8:24. doi: 10.1186/1741-7007-8-24 (PMC2859859; doi:10.1186/1741-7007-8-24)
Supplement: Additional file 1 — Supplementary information. Figure S1, discussion of Figure S1, and Table S1. [file 1741-7007-8-24-S1.PDF]

**BMC Biology**

# Supplementary Information

# Denitrification in Human Dental Plaque

Frank Schreiber, Peter Stief, Armin Gieseke, Ines M. Heisterkamp, Willy Verstraete,

Dirk de Beer and Paul Stoodley

**Contents**

Supplementary Figure S1
Supplementary Discussion to Figure S1
Supplementary Table S1
Supplementary References

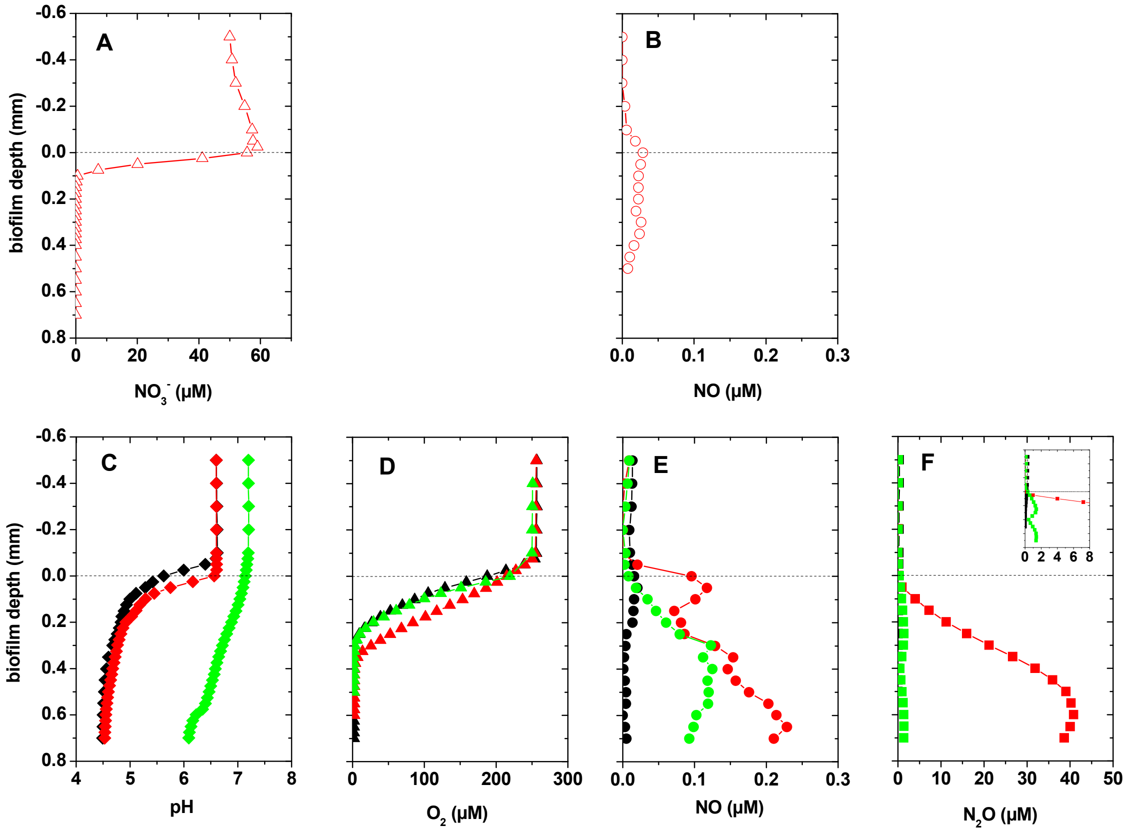


**Figure S1.** *In situ* concentration micro-profiles measured in a dental biofilm from a different individual as presented in Figure 2. The colour code and the setup of the figure are directly comparable to Figure 2. The medium contained a non-buffered mineral mix and 2% sucrose. The upper panels **(A-B)** show measurements with 50 µM $NaNO_3$ in the overlying medium. The lower panels **(C-F)** show measurements in the absence of $NaNO_3$ (black symbols) and in the presence of 760 µM $NaNO_3$. (red and green symbols). Measurements depicted by the green symbols were performed in the presence of 1x phosphate-buffered saline (pH 7.2) and 760 µM $NaNO_3$. The inset in panel F shows the same data with magnification of the x-axis. The horizontal line represents the biofilm surface. Measurements were done in the same sample spot and are thus directly comparable.

**Supplementary Discussion to Figure S1**

The data shown in Figure S1 are derived from the same type of experiment, but with plaque from a different individual as is shown and discussed in the paper in Figure 2. Even though both samples display different micro-environmental conditions for the various solutes, the characteristics of the turnover dynamics are similar in both samples. In the following, we will assess if each effect that we discuss in the paper (here in italics) for the sample depicted in Figure 2 is also present in the sample depicted in Figure S1.

*Microsensor measurements showed that* $NO_3^-$ *was consumed in the presence of* $O_2$ *and that the denitrification intermediates NO and* $N_2O$ *were formed at the same time (Figure 2A-D).*

Figure S1D shows that $O_2$ is depleted at 0.4 mm (red symbol) or 0.3 mm (green and black symbols) inside the biofilm. Exposing the same sample to 50 µM $NO_3^-$ shows that $NO_3^-$ is completely consumed within the first 0.1 mm inside the biofilm (Figure S1A). Thus, $NO_3^-$ is consumed in the presence of $O_2$. In contrast to the measurements shown in Figure 2, 50 µM $NO_3^-$ are limiting, which indicates that $NO_3^-$ assimilation and denitrification are not at their maximum capacity in this plaque sample. However, Figure S1B, E and F show that the denitrification intermediates NO and $N_2O$ are present under oxic conditions.

$NO_3^-$ *was the source for NO and* $N_2O$ *in dental biofilms. This was shown by NO and* $N_2O$ *formation being restricted to the presence of* $NO_3^-$ *(Figure 2G and H).*

Figure S1E and F also show that production of NO and $N_2O$ is restricted to the presence of $NO_3^-$ in the overlying medium (compare black symbols without $NO_3^-$ to red and green symbols with $NO_3^-$).

*In non-buffered medium, bacterial activity decreased biofilm pH < 5 (Figure 2E) and depth-averaged NO concentrations increased from 0.08 to 0.15 µM (Figure 2G).*

Figure S1C also shows that bacterial activity decreased plaque pH < 5 and that buffering the medium abolished this effect. Figure S1E and F show that NO and $N_2O$ concentrations in the presence of $NO_3^-$ are higher under non-buffered than buffered conditions. In the plaque sample displayed in Figure S1E the depth-averaged NO concentration increased from 0.105 µM to 0.162 µM, which is in a similar range than the increase from 0.08 to 0.15 µM (Figure 2G). Comparing buffered and non-buffered conditions shows that the increase of $N_2O$ was more pronounced than the increase of NO.

*Oxygen uptake in the presence of $NO_3^-$ was higher at neutral pH than under acidic conditions (Figure 2E and F). The $O_2$ profiles showed that the flux of $O_2$ decreased by 50 %, namely from -105 nmol $cm^{-2}$ $h^{-1}$ under buffered conditions to -43 nmol $cm^{-2}$ $h^{-1}$ under non-buffered conditions. Acidic pH alone did not lead to reduced $O_2$-uptake when $NO_3^-$ was absent, as the $O_2$-flux was -143 nmol $cm^{-2}$ $h^{-1}$.*

Figure S1D shows that the $O_2$-uptake in the absence of $NO_3^-$ (-833 nmol $cm^{-2}$ $h^{-1}$) was in the same range than $O_2$-uptake in the presence of $NO_3^-$ and buffer (-1044 nmol $cm^{-2}$ $h^{-1}$). In contrast, $O_2$ uptake in the presence of $NO_3^-$ under acidic conditions (-402 nmol $cm^{-2}$ $h^{-1}$) was reduced by 62 % as compared to the uptake under buffered conditions. This compares well with the 50% reduction of the $O_2$ uptake in the plaque presented in Figure 2, even though the total $O_2$ uptake dynamics are different in both samples.

**Supplementary Table S1.** PCR detection of denitrification genes in dental plaque

| Target | Primer | Primer sequence 5'-3' | Positive Control | Reference |
|---|---|---|---|---|
| ***narG*** | narG1960f | TAYGTSGGSCARGARAA | *Castellaniella defragrans* | 1[a] |
| | narG2650r | TTYTCRTACCABGTBGC | | |
| ***nirS***[b] | nirS1F | CCTAYTGGCCGCCRCART | *Pseudomonas stutzeri* DSM 5190 | 2 |
| | nirS6R | CGTTGAACTTRCCGGT | | |
| | cd3aF | GTSAACGTSAAGGARACSGG | | 3 |
| | R3cd | GASTTCGGRTGSGTCTTGA | | 4 |
| ***nirK***[c] | nirK1F | GGMATGGTKCCSTGGCA | *Hyphomicrobium denitrificans* DSM 1869 | 2 |
| | nirK5R | GCCTCGATCAGRTTRTGG | | |
| | F1aCu | ATCATGGTSCTGCCGCG | | 5 |
| | R3Cu | GCCTCGATCAGRTTGTGGTT | | |
| ***qnorB*** | qnorB2F | GGNCAYCARGGNTAYGA | *Castellaniella defragrans* | 6 |
| | qnorB7R | GGNGGRTTDATCADGAANCC | | |
| ***cnorB*** | cnorB2F | GACAAGNNNTACTGGTGGT | *Pseudomonas stutzeri* DSM 5190 | 6 |
| | cnorb7R | TGNCCRTGNGCNGCNGT | | |
| ***nosZ*** | nosZ1181F_a | CGCTGTTCMTCGACA GYCAR | *Pseudomonas stutzeri* DSM 5190 | 7 |
| | nosZ1880R | ATGTGCAKIGCRTGGCAGAA | | |

[a] modified: 10 min at 95°C, touch-down PCR: 38 cycles of 1 min at 94°C, 1 min annealing, 1 min at 72°C, annealing temperature decreased with 0.5°C/cycle from 59°C to 52°C, final elongation for 6 min at 72°C

[b] product from nirS1F/nirS6R PCR reaction was used as template for PCR with cd3aF/R3cd

[c] product from nirK1F/nirK5R PCR reaction was used as template for PCR with F1aCu/R3Cu

## Supplementary References


1. Philippot, L., Piutti, S., Martin-Laurent, F., Hallet, S. & Germon, J.C. Molecular analysis of the nitrate-reducing community from unplanted and maize-planted soils. *Appl. Environ. Microbiol.* **68**, 6121-6128 (2002).
2. Braker, G., Fesefeldt, A. & Witzel, K.P. Development of PCR primer systems for amplification of nitrite reductase genes (nirK and nirS) to detect denitrifying bacteria in environmental samples. *Appl. Environ. Microbiol.* **64**, 3769-3775 (1998).
3. Michotey, V., Mejean, V. & Bonin, P. Comparison of methods for quantification of cytochrome cd(1)-denitrifying bacteria in environmental marine samples. *Appl. Environ. Microbiol.* **66**, 1564-1571 (2000).
4. Throback, I.N., Enwall, K., Jarvis, A. & Hallin, S. Reassessing PCR primers targeting nirS, nirK and nosZ genes for community surveys of denitrifying bacteria with DGGE. *FEMS Microbiol. Ecol.* **49**, 401-417 (2004).
5. Hallin, S. & Lindgren, P.E. PCR detection of genes encoding nitrile reductase in denitrifying bacteria. *Appl. Environ. Microbiol.* **65**, 1652-1657 (1999).
6. Braker, G. & Tiedje, J.M. Nitric oxide reductase (norB) genes from pure cultures and environmental samples. *Appl. Environ. Microbiol.* **69**, 3476-3483 (2003).
7. Stief, P., Poulsen, M., Nielsen, L.P., Brix, H. & Schramm, A. Nitrous oxide emission by aquatic macrofauna. *Proc. Natl. Acad. Sci. U. S. A.* **106**, 4296-4300 (2009).
